# Historical and Contemporary DNA Indicate Fisher Decline and Isolation Occurred Prior to the European Settlement of California

**DOI:** 10.1371/journal.pone.0052803

**Published:** 2012-12-26

**Authors:** Jody M. Tucker, Michael K. Schwartz, Richard L. Truex, Kristine L. Pilgrim, Fred W. Allendorf

**Affiliations:** 1 United States Department of Agriculture Forest Service, Sequoia National Forest, Porterville, California, United States of America; 2 United States Department of Agriculture Forest Service, Rocky Mountain Research Station, Missoula, Montana, United States of America; 3 Wildlife Biology Program, University of Montana, Missoula, Montana, United States of America; 4 United States Department of Agriculture Forest Service, Rocky Mountain Region, Golden, Colorado, United States of America; 5 Division of Biological Sciences, University of Montana, Missoula, Montana, United States of America; University of Guelph, Canada

## Abstract

Establishing if species contractions were the result of natural phenomena or human induced landscape changes is essential for managing natural populations. Fishers (*Martes pennant*i) in California occur in two geographically and genetically isolated populations in the northwestern mountains and southern Sierra Nevada. Their isolation is hypothesized to have resulted from a decline in abundance and distribution associated with European settlement in the 1800s. However, there is little evidence to establish that fisher occupied the area between the two extant populations at that time. We analyzed 10 microsatellite loci from 275 contemporary and 21 historical fisher samples (1880–1920) to evaluate the demographic history of fisher in California. We did not find any evidence of a recent (post-European) bottleneck in the northwestern population. In the southern Sierra Nevada, genetic subdivision within the population strongly influenced bottleneck tests. After accounting for genetic subdivision, we found a bottleneck signal only in the northern and central portions of the southern Sierra Nevada, indicating that the southernmost tip of these mountains may have acted as a refugium for fisher during the anthropogenic changes of the late 19^th^ and early 20^th^ centuries. Using a coalescent-based Bayesian analysis, we detected a 90% decline in effective population size and dated the time of decline to over a thousand years ago. We hypothesize that fisher distribution in California contracted to the two current population areas pre-European settlement, and that portions of the southern Sierra Nevada subsequently experienced another more recent bottleneck post-European settlement.

## Introduction

Over the past 100 years there has been a marked reduction in many species geographic ranges. For rare or hard to observe species, it is often unclear if their absence is a response to a changing landscape, or if they have been absent from an area for an extended period of time. If they were considered present early during the last epoch, but are now unable to be detected, this is seen as a natural range contraction [1]. On the other hand, if they were considered present until the last century, but are now unable to be detected, this is often viewed as caused by human induced disturbances. Establishing if contractions of species were the result of natural causes or human-induced landscape changes is essential for managing natural populations. Mistakes associated with misidentifying the geographic range of a species and misattributing declines in geographic range can have large effects on the allocation of scarce conservation resources [2].

Traditionally, the historical distribution of a species has been based on accounts of explorers, naturalists, and indigenous peoples that are verified by specimens preserved in museum collections. Recently, technological and laboratory advances in molecular genetics have created the ability to extract DNA from historical specimens and examine the population genetic signals obtained, providing a new tool by which we can test ideas proposed by these early naturalists [3], [4]. Historical and contemporary genetic information can provide insight into the nature of population expansions or declines [5], [6], the loss of genetic diversity [7], [8], temporal changes in population connectivity [9], or the historical range of a species [10], [11].

Prior to European settlement, fishers (*Martes pennanti*) were distributed widely in both Canada and the northern U.S. forests [12]. In the late 1800's and early 1900's, fisher populations dramatically declined due to a combination of fur trapping, logging, and predator control and by the early 1900's were extirpated from large portions of their historic range [13]. Reintroductions and expansions from refugia populations have been successful in reestablishing fisher populations in the eastern and Rocky Mountain states [14]–[18]. However, West Coast populations have not experienced the same degree of recovery. There are 5 geographically disjunct fisher populations present on the West Coast: two native populations in California [19], [20], a reintroduced population established in the 1950's in Oregon, and two recently reintroduced populations (one on the Olympic Peninsula in Washington State and one in California [21], [22]).

**Figure 1:**
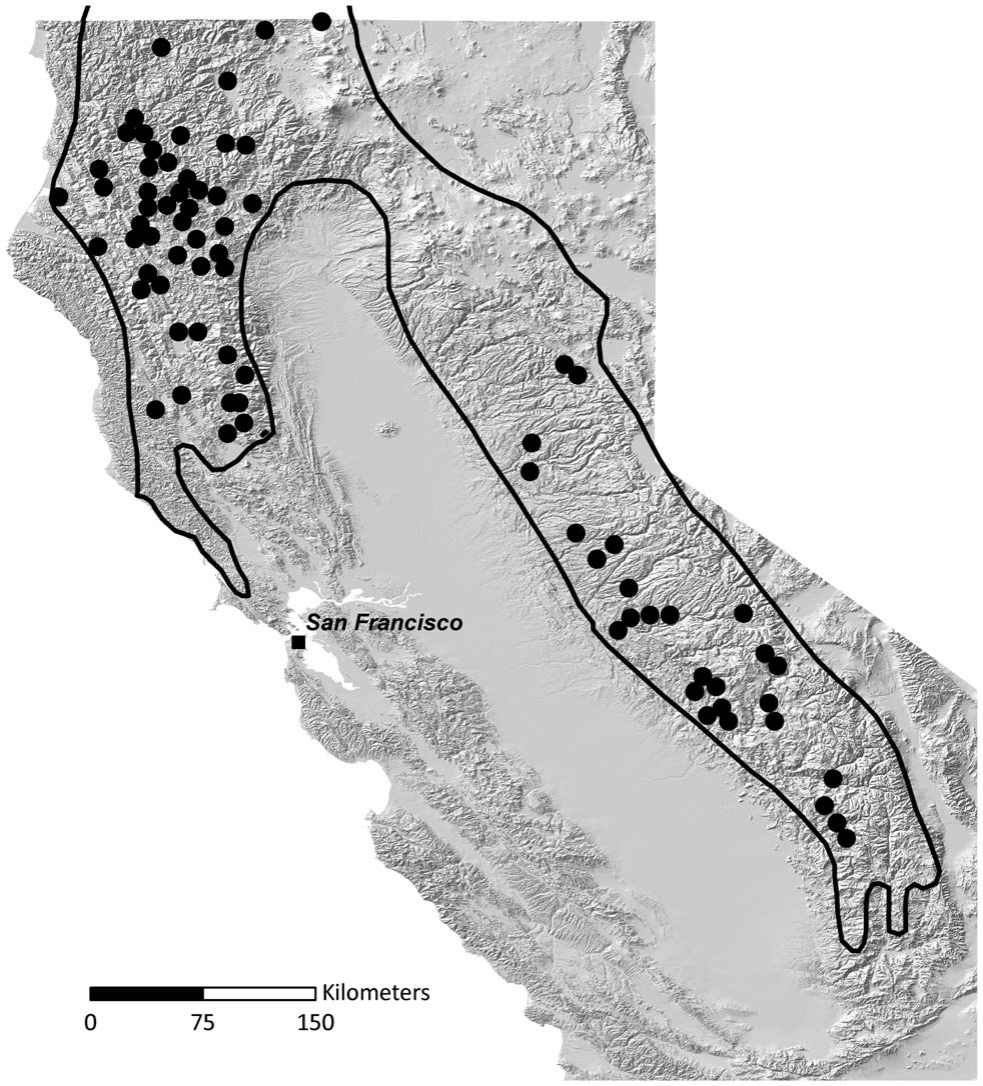
Historical range map for fisher in California. Fisher locations used by Grinnell et al. [26] to document the distribution of fisher in California. Locations are based primarily on reports of trappers and collecting expeditions from 1919–1924. Grinnell wrote that “spots [black dots] indicate, almost all of them with certainty, the locality of capture; probably some indicate the residence of post office or trapper”. The outlined area is the Grinnell et al. [26] assessment of the range of fisher in California from ∼1850–1925.

The two native fisher populations in California are geographically and genetically isolated [19], [23], [24]. Conservation concerns are particularly acute for fisher in the southern Sierra Nevada Mountains because its population size is estimated at less than 300 adults [25]. The majority of information about the history of fisher in California comes from the work of the naturalist Joseph Grinnell. Grinnell et al. [26] used information from extensive surveys, collecting expeditions, trapping records, and local knowledge from approximately 1910–1930 to create distribution maps for 21 species of carnivores. Grinnell's range maps show the historical fisher range as continuous from the northwestern Klamath and Siskiyou Mountains to the southern tip of the Sierra Nevada (Fig. 1).

Fisher populations in California are thought to have declined precipitously in both abundance and distribution over the last 150 years due to habitat alteration and fur trapping associated with the European settlement of California beginning with the gold rush in 1848 [27]. Currently, the two areas that maintain native populations of fisher in California are separated by a 420 km gap, which is more than four times the maximum dispersal distance of fisher [19], [27]. The reason for this gap is not well understood. The majority of habitat in this area is contiguously forested and appears, at least superficially, to be suitable for fisher occupancy. Grinnell's range map shows only a few records of fisher in the central Sierra Nevada and none in the northern Sierra Nevada (Fig. 1), but despite these facts this gap is considered part of the historical range of the species [26].

The accepted hypothesis for the lack of records in the gap area is that the northern and central Sierra Nevada had experienced a greater degree of anthropogenic change at the time of the Grinnell surveys than the southern Sierra Nevada and that the species was already extirpated from the gap by the early 1900's [27]. The central and northern Sierra was the main area of human development as a result of the gold rush. Yet, in a study of the history of forest conditions in the Sierra Nevada, McKelvey and Johnston [28] found that due to transportation limitations, logging at the turn of the century was relatively limited in the central and northern Sierra. At this time even the most heavily affected National Forest in this area still had 50% virgin forest and therefore, likely retained areas of large trees that are associated with fisher habitat in California [29]–[31]. Based on such information, it is unclear why fisher would have been completely extirpated from the gap prior to Grinnell's surveys.

An alternative hypothesis is that this distributional gap may not be the result of recent human influences but rather is a historical discontinuity in fisher distribution that existed prior to the European settlement of California. Fishers are thought to have colonized the West Coast of the United States in a relatively recent range expansion from British Columbia southward in a series of stepwise founder events during the mid to late Holocene [12], [23], [32]. Evidence of an early peninsular expansion is found in the gradient of genetic diversity decreasing from north to south down the West Coast [23], and the existence of a shared haplotype between British Columbia and a historical sample from northwestern California [32]. However, evidence indicates there has been little gene flow between the two regions in the time since colonization with high genetic divergence in nuclear DNA (*F*_ST_ = 0.48–0.60) and the absence of a shared mtDNA haplotype between northwestern California and the southern Sierra Nevada [23], [24].

There are important conservation concerns regarding the southern Sierra Nevada fisher population's risk of extinction stemming from its small population size, isolation, and low genetic diversity. Determining whether the isolation of fisher in the southern Sierra Nevada has occurred recently (within the last 150 years), or if the population has been persisting in long-term isolation, are important alternative hypotheses that need to be distinguished to inform future conservation decisions. Discussions of how to manage this population to support long-term persistence have included the potential need for translocations to augment populations or reintroductions into the current gap region to re-establish connectivity [33], [34]. If population decline and isolation occurred recently then potential risk from inbreeding depression due to small population size may be an important consideration for the southern Sierra Nevada and aggressive measures to restore genetic connectivity may in fact be prudent. Conversely, detection of a more ancient timeline for isolation would indicate the potential for significant local adaptations within the population and that creating genetic connectivity with northwestern California fishers could actually trigger a reduction in fitness due to outbreeding depression [35], [36].

Recent research has attempted to address the historical continuity of fisher populations in California using mtDNA. Knaus et al. [24] sequenced the entire mtDNA genome for 40 fisher samples and found the southern Sierra Nevada to be fixed for a single haplotype that is different from the closest haplotype in northwestern California by 9 base-pair substitutions. The absence of a shared mtDNA haplotype between northwestern California and the southern Sierra Nevada and the amount of genetic differentiation between haplotypes indicates long term isolation. Using a molecular clock approach, they estimated the divergence between these two populations occurred thousands of years ago [24].

While the results of Knaus et al. [24] are striking, mtDNA is maternally transmitted and consequently only provides insight into female mediated gene flow. This may be especially problematic for species such as fisher that exhibit female philopatry where most of the large movements are made by males [37]. This would result in primarily male mediated gene flow across long distances. As nuclear DNA is bipaternally inherited, it may show different genetic signals from mtDNA that reflect the influence of males on connectivity. Numerous studies have shown discord between estimates of divergence from mtDNA versus nuclear DNA and emphasized the importance of analyzing both mtDNA and nuclear DNA prior to making conservation decisions [38]–[40].

Our objective is to use nuclear DNA to distinguish between the alternate hypotheses that the geographic isolation of the two California fisher populations occurred before or after the European settlement of California. We also wish to more precisely date this divergence. The hypothesis that fisher decline and isolation in California occurred prior to 1850 would be supported by lack of evidence of a recent bottleneck and contraction in population size greater than 160 years ago. Conversely, if the hypothesis that isolation occurred after 1850 is correct, we would expect to see evidence of a recent population bottleneck and a contraction in population size within the last ∼160 years. Evidence of post-European isolation would be at odds with mtDNA analyses [24] and indicate male mediated gene flow between California fisher populations. In a broader sense, this research is also aimed at showing the importance of understanding historical biogeographic patterns to better understand and manage contemporary patterns of species on the landscape.

## Materials and Methods

### Ethics Statement

All necessary permits were obtained for the described field studies. These included a Scientific Research and Collecting Permit from the U.S. Department of the Interior, National Park Service (SEKI-2008-SCI-0014).

### Samples

**Figure 2:**
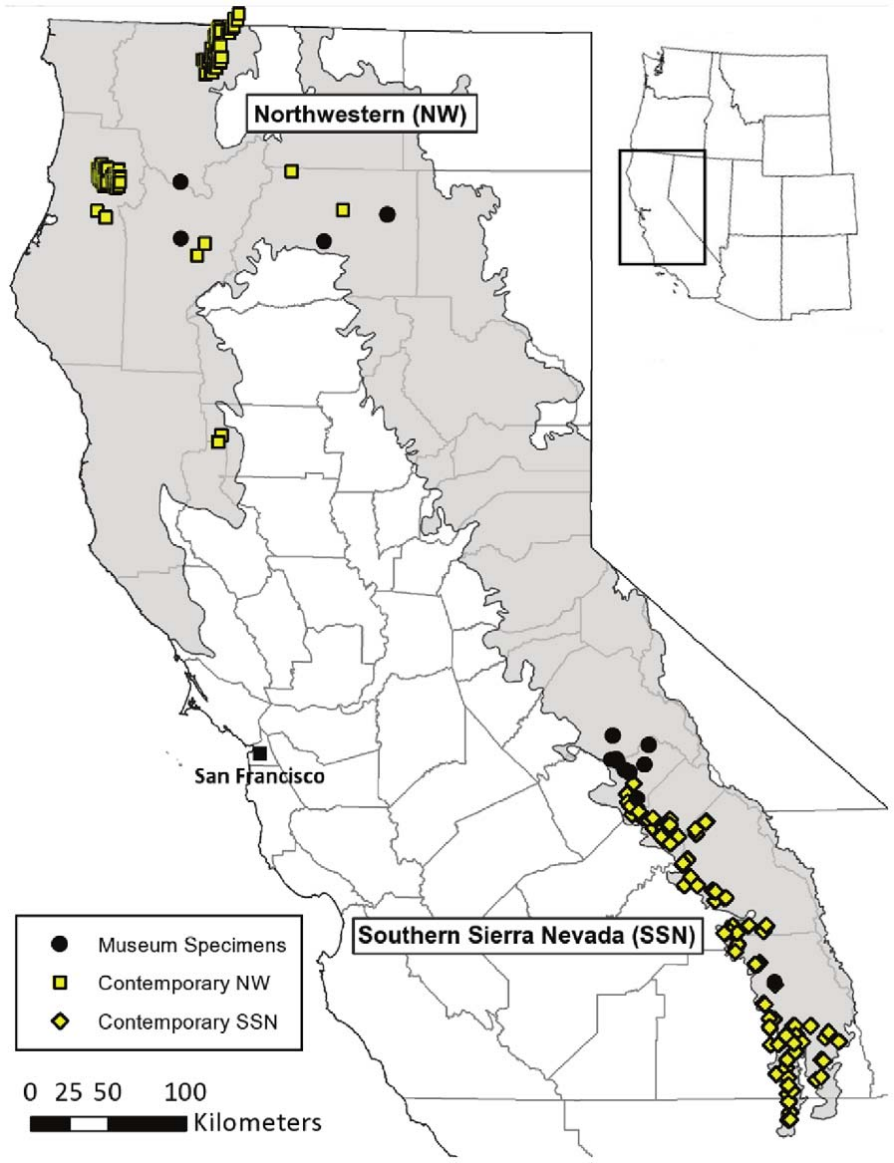
Sample locations. Locations of the historical (H) and contemporary (C) genetic samples from the northwestern mountains (NW) and southern Sierra Nevada (SSN) of California. Sample size is as follows: NW_H_ n = 5, SSN_H_ n = 16, NW_C_ n = 148, SSN_C_ n = 127. Grinnell's assumed historical range as adapted by Davis et al. [115] is shown in gray.

We obtained both historical (H) and contemporary (C) genetic samples from the extant range of fisher in California which includes one area in northwestern California (NW) and a second area in the southern Sierra Nevada (SSN) (Fig. 2). The NW and SSN populations were defined *a priori* based on previous research that indicated that these populations are geographically isolated due to an unoccupied 420 km gap between them [19], [27], as well as genetically isolated [23], [24]. We genotyped 127 individuals from hair samples collected in the SSN_C_ through the U.S. Forest Service Sierra Nevada Carnivore Monitoring Program [41]. In the NW_C_ we obtained genotypes from 148 individuals based on hair, scat, and tissue samples collected in collaboration with a number of existing research projects in the region. Genetic samples from both regions were collected from 2006–2009. Historical samples were located by searching databases of museum collections. We found 41 fisher specimens from 1884–1920 in the collections of the Smithsonian National Museum of Natural History and the Museum of Vertebrate Zoology at the University of California, Berkeley (Table S1). We collected maxilloturbinal bones from inside the nasal cavity to maximize the probability of obtaining high quality DNA while minimizing damage to specimens [42], [43]. We also collected tissue from pelts, bone fragments, or muscle when available. In total, 17 historical specimens were obtained from the NW_H_ and 24 from the SSN_H_. We did not find any historical fisher specimens from the current gap in fisher distribution.

### Laboratory Analysis

We extracted DNA from museum specimens in a separate laboratory used exclusively for the extraction and processing of genetic material from museum specimens following recommended ancient DNA protocols [42], [44]. We analyzed the samples at 10 microsatellite loci. *MP0059*, *MP0144*, *MP0175*, *MP0197*, *MP0200*, and *MP0247* were developed from tissue samples from the SSN [45]. Loci *MA1* [46], *GGU101*, *GGU216* [47], and *LUT733* [48], were developed in other mustelid species [marten (*Martes americana*), wolverine (*Gulo gulo*), and otter (*Lutra lutra*), respectively].

The quality and quantity of DNA obtained from historical and non-invasive samples can vary considerably because of age and different methods of preservation and storage. The potential for degraded or low quantity DNA increases the likelihood of genotyping errors such as allelic dropout or false alleles [49]. To address this potential for error, we ran samples a minimum of three times per locus and accepted genetic data only if the samples produced consistent genotype scores [50], [51]. If the genotype differed in one or more of these amplifications, we conducted an additional round of 3 amplifications. If multiple inconsistencies were found in the genotype at a locus we removed that sample from the analysis. We also checked for genotyping errors using the software DROPOUT [52].

### Statistical analyses

We tested microsatellite genotypes for departures from Hardy-Weinberg proportions at each locus and gametic disequilibrium for each pair of loci using Fisher's exact test in Genepop 4.0 [53], [54]. We also used Genepop 4.0 to calculate expected heterozygosity (*H*_E_), proportional excess of homozygotes (*F*_IS_), *F*_ST_ [55], *R*_ST_ [56], and conduct tests for genetic differentiation between sample groups. The amount of genetic diversity present in the sample groups was compared using paired t-tests of arcsine-transformed *H*_E_, and *A*_R_ [57]. We used sequential Bonferroni corrections to correct for multiple comparisons when assessing statistical significance [58].

### Detecting bottlenecks

We used three methods to determine whether fisher in California had experienced a recent reduction in population size. We first tested for heterozygosity excess which is characteristic of bottlenecked populations using BOTTLENECK 1.2.02 [59]. This heterozygosity excess exists because rare alleles are lost more rapidly during a bottleneck but have little impact on heterozygosity [60]. Heterozygosity excess is transient and will only persist for 0.2 – 4N_e_ generations after the bottleneck. The average expected heterozygosity at mutation-drift equilibrium was calculated using 5000 replications assuming a two-phase mutational model. We conducted analyses with both 5% and 20% of mutations set as multistep mutations in the two-phase model with a variance of 12 to encompass the range of multistep mutations observed in natural populations [61]. The observed heterozygosity was then tested against the equilibrium expected heterozygosity using the Wilcoxon signed-rank test. We also conducted the test excluding all loci that were out of Hardy-Weinberg, as such loci can create bias, but doing so did not significantly change the results.

Second, we also used BOTTLENECK to test for a shift in the mode of the distribution of allele frequencies. This mode shift distortion is transient and can only be detected for a few dozen generations. Luikart et al. [62] found using simulations that the graphical mode shift method is likely (P>.80) to detect a bottleneck of up to 20 breeding individuals using 8–10 microsatellite loci. The mode shift test could not be applied to the historical samples because at least 30 individuals are needed to avoid high type 1 error rates.

The third method used detects reductions in effective population size (*N*_e_) using the *M*-Ratio which is defined as *M* = k/r where k is the total number of alleles and r is the range in allele size [63]. Because a bottleneck causes a greater reduction in the number of alleles than in the range of allele sizes, *M* is smaller in reduced populations. Garza and Williamson [63] found that a reduction in population size can be detected using *M* for 125 generations if the population rebounded quickly in size or 500 generations if the population remained reduced. We used the software *M*_P_Val to calculate *M* and the software *M*_Critical to determine the cutoff value for statistical significance [63]. We set model parameters at 90% single-step mutations and 10% multi-step mutations (p_s_) and the average size of multistep mutation (Δg) of 3.5 with the mutation rate μ held constant at 5×10^−4^. In this model θ = 4*N*_e_μ so if μ is held constant different values of θ are representative of different starting (pre-decline) *N*_e_. As the equilibrium *N*_e_ for fisher in California is not known, we calculated *M* and *M*-Critical values for four different values of θ (1, 2, 5, and 10) which represent a wide range of pre-decline *N*_e_ (500, 1000, 2500, and 5000 respectively).

**Figure 3:**
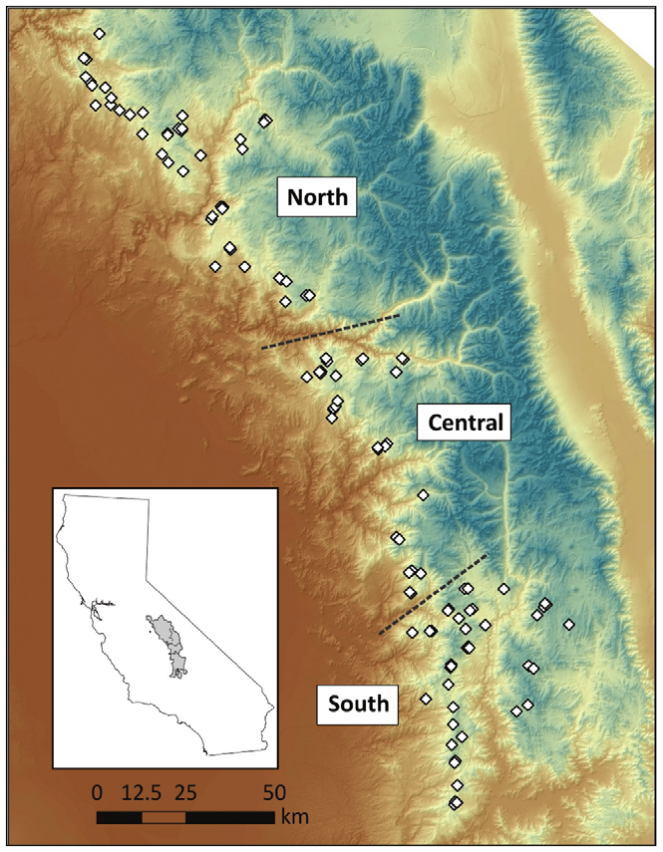
Population subdivision in the southern Sierra Nevada. Approximate location of population subdivisions used in bottleneck analyses within the contemporary southern Sierra Nevada.

The presence of unaccounted for genetic subdivision has the potential to bias bottleneck tests [64]. While genetic subdivision has not been previously detected in the NW_C_ population, past research has shown significant subdivision in the SSN_C_ [23]. To assess the influence of this subdivision, we divided the SSN_C_ into three genetic groups and assessed the influence of this on the bottleneck tests. The subdivisions between demes in the SSN_C_ roughly correspond to the areas north of the Kings River (North), between the Kings River and Middle Fork of the North Fork of the Tule River (Central), south of the Middle Fork of the North Fork Tule River (South) (Fig. 3). Previous research on fisher populations in southern Ontario has found rivers to be a major barrier to genetic connectivity [18], [65]. These subdivision boundaries are also supported by data from a recent population genetic analysis of the SSN_C_ showing moderate subdivision (*F*_ST_ 0.05–0.13) between these areas (J.M. Tucker unpublished data).

### Demographic history models

We employed a coalescent-based Bayesian analysis to assess the most recent major change in *N*_e_ and to estimate the date of the change. This model assumes that an ancestral *N*_e_ (*N*_1_) changed to the current *N*_e_ (*N*_0_), at a time *T* generations ago [66], [67]. This model uses a stepwise mutation model and assumes a mutation rate scaled in terms of the current populations size such that that θ = 2*N*_0_μ, where μ is the per locus mutation rate. While this method does employ a strict stepwise mutation model, it has been found to be robust to moderate departures created by the presence of multistep mutations [68]. The method then estimates the posterior distributions of *N*_1_, *N*_0_, *T*, and θ that describe the genealogical and demographic history of the sample, assuming either linear or exponential size change. Prior distributions for *N*_1_, *N*_0_, *T*, and θ are assumed to be log normal with their means and standard deviations drawn from hyperprior distributions truncated at zero. We conducted the analysis using MSVAR 1.3 [67] which uses Markov Chain Monte Carlo (MCMC) simulations to estimate the posterior distribution of each parameter.

We conducted 6 independent simulations of the model varying the prior and hyperprior distributions with a range of biologically realistic distribution values to examine their effect on the posterior distributions. These variations of the priors had little effect on the posterior distribution of the models so prior distributions for all other analyses were set to the parameters of simulation 1 (Table S2). To check for the convergence of model we conducted five replications of the simulations for each data set. Each simulation was performed for 2×10^9^ iterations with parameter values recorded every 1×10^5^ iterations resulting in 20,000 records.

We removed the first 10% of data from each chain as burn-in and assessed chain convergence using the Brooks, Gelman, and Rubin Convergence Diagnostic test [69], [70]. We conducted convergence diagnostics in R version 2.11.1 [71] using the package BOA version 1.1.7 [72]. The test statistic is a multivariate potential scale reduction factor (MPSRF) that assesses the convergence of a set of parameters simultaneously. The MPSRF value for all parameters was ∼1.0 indicating acceptable chain convergence. We then combined the last 50% of the data from each chain (10,000 records/chain, 50,000 total records) and calculated the mode and 90% highest posterior densities (HPD) of the posterior distributions of each parameter using the R-package Locfit 1.5–6 [73]. We evaluated the strength of evidence for population expansion versus decline by calculating the Bayes factor for each of the models [74], [75] as described by Storz and Beaumont [67]. The Bayes factor indicates the following levels of support for the model; BF<0.33 = false detection of contraction/expansion, 0.33–3 = no support, 3–10 = substantial support, and ≥10 = strong support [74].

While the generation time (average age of reproduction) for fisher has not been well studied, the average age of first reproduction is estimated at 2–3 years, with high reproductive rates documented in 5–7 year old females [76], and successful reproduction found in females as old as 10 years (C. Thompson personal communication). We used a generation interval of five years. Parameter estimates of *T* can easily be adjusted for different generation times by multiplying accordingly. We ran the simulations for all data sets using both the exponential and linear models.

## Results

We successfully obtained genotypes at a minimum of seven loci for 127 individuals in the SSN_C_, 148 individuals in the NW_C_, 16 individuals from the SSN_H_, and five individuals from the NW_H_ (Table 1). The dates of the historical samples that successfully yielded microsatellite genotypes ranged from 1884–1920, which represents the overall timeframe of available historical samples (Table S1). Nine of the 10 microsatellite loci were polymorphic in all samples. The exception was the *MA1* locus which was monomorphic in the NW_C_. Tests for Hardy-Weinberg proportions showed deviation from expected values at *MP200* and *MP59* in the SSN_C_. However, these deviations are non-significant after accounting for genetic population structure. We also found *MP200* deviated in the SSN_H_ to have a homozygote excess compared to expected Hardy-Weinberg proportions. To assess the influence of this locus, we conducted SSN_H_ analyses both with and without this locus but did not find any notable difference in results.

**Table 1:** Estimates of genetic diversity for the northwest (NW) and southern Sierra Nevada (SSN) at 10 microsatellite loci: sample size (n), expected heterozygosity (*H*_E_), proportional excess of homozygotes (*F*_IS_), mean number of alleles (*A*), and allelic richness (*A*_R_).

	n	*H*_E_	*F*_IS_	*A*	*A*_R_
NW-Historical	5	0.635	0.028	3.60	3.34
NW-Contemporary	148	0.431	0.028	3.75	2.17
SSN-Historical	16	0.590	0.046**	3.60	2.81
SSN-Contemporary	127	0.565	0.101***	3.50	2.51

Allelic richness is based on a minimum size of 4 individuals which represents the number of individuals with genotypes at all 10 loci in the historical NW sample.

** P<0.05;

*** P<0.001.

While we did not find any evidence for departure from Hardy-Weinberg proportions at individual loci, we did find some important patterns over all loci within each sample group. *F*_IS_ values were small and statistically insignificant in both the NW_H_ and NW_C_ samples, but had significant p-values in both the SSN_H_ and SSN_C_. Most notably, the SSN_C_ showed a large deficit of heterozygotes (*F*_IS_ = 0.101, p<0.001) (Table 1). This is indicative of the potential presence of the Wahlund effect [77] in the SSN, in which unaccounted for population subdivision in a sample generates a deficit of heterozygotes relative to expected Hardy-Weinberg proportions.

Tests for gametic disequilibrium did not find any strong associations between loci. After correcting for multiple comparisons statistically significant gametic disequilibrium was found between two pairs of loci in the SSN_C_ (*MP197*/*MP200*, and *MA1*/*MP144*), one pair in the NW_C_ (*MP175*/*LUT733*), and none in either historical sample group. No pairs of loci were consistently significant across sample groups indicating that the loci used were assorting independently.

We did not find any difference in the amount of genetic diversity within sample groups with paired t-tests showing no significant differences in *H*_E_, or *A*_R_. However, all metrics of genetic diversity were lowest in the NW_C_ (Table 1). *H*_E_ was markedly lower in the NW_C_ (0.431) compared to all other samples (0.57–0.64). Allelic richness (*A*_R_) was higher in both historical samples (NW_H_ = 3.34, SSN_H_ = 2.81) than in either contemporary sample (NW_C_ = 2.17, SSN_C_ = 2.51). Samples in the NW_C_ were monomorphic at locus MA1, and have extremely low diversity at the MP200 locus (2 of 3 alleles at 1% frequency). When these two loci were removed from calculations the NW_C_ *H*_E_ increases to 0.54 which is similar to the value for the other sample groups at 8 loci (NW_H_ = 0.55, SSN_H_ = 0.60, SSN_C_ = 0.55) and *A*_R_ in the two contemporary populations becomes equal (SSN_C(8loci)_ = NW_C(8loci)_ = 2.46).

We found each group to be significantly genetically different. Tests for genic differentiation between sample groups were significant at P<0.001. *F*_ST_ and *R*_ST_ values were moderate between historical sample groups (NW_H_/SSN_H_: *F*_ST_ = 0.10, *R*_ST_ = 0.20) but increased over time with contemporary samples showing increased divergence (SSN_C_/NW_C_: *F*_ST_ = 0.37, *R*_ST_ = 0.58). We also found temporal divergence over time with moderate *F*_ST_ values between temporally spaced samples in the same geographic location (SSN_H_/SSN_C_ = 0.17, NW_H_/NW_C_ = 0.20) (Table 2). *R*_ST_ values were considerably higher than *F*_ST_ values indicating that when variation in allele length is accounted for genetic divergence between samples groups is even greater.

**Table 2:** Pairwise comparisons of genetic differentiation between samples with *R*_ST_ above the diagonal and *F*_ST_ below the diagonal.

	NW_H_	NW_C_	SSN_H_	SSN_C_
NW_H_	–	0.321	0.195	0.500
NW_C_	0.198	–	0.363	0.581
SSN_H_	0.098	0.291	–	0.265
SSN_C_	0.208	0.374	0.170	–

H denotes historical samples and C denotes contemporary samples. All pairwise comparisons shown in the table are significant at P<0.01.

### Population bottlenecks

We did not find any signal of a recent population bottleneck for either the historical or contemporary NW samples. Both NW samples had non-significant results for the Wilcoxon heterozygosity excess test and the NW_C_ was also negative for the shifted mode test. Bottleneck tests for the SSN were mixed. For the SSN_C_ the heterozygosity excess test was statistically significant regardless of the proportion of multistep mutations in the two-phase model (5%: p = 0.04, 20%: p<0.001), but showed no evidence of a shifted mode. The SSN_H_ was significant at α = 0.05 for heterozygosity excess but only when using 20% multistep mutations (5%: p = 0.10, 20%: p = 0.05). We found no evidence of a population bottleneck for any sample group using the *M*-Ratio method (Table 3).

**Table 3:** Results of BOTTLENECK tests including the p values for the Wilcoxon heterozygosity (*H*_E_) excess test with two different proportions of multistep mutations in the two phase model (TPM), shifted mode test, and *M*-Ratio value and *M*-Critical values.

	*n*	*H*_E_ Excess TPM 20%	*H*_E_ Excess TPM 5%	Shifted Mode	*M*-Ratio	*M*-Critical θ = 1	*M*-Critical θ = 10
NW_H_	5	0.19	0.22	-	0.92	0.71	0.55
NW_C_	148	0.08	0.22	No	0.91	0.77	0.71
SSN_H_	16	0.05	0.10	-	0.87	0.78	0.64
SSN_C_	127	0.00	0.04	No	0.89	0.78	0.72
SSN_C_ – North	44	<0.001	<0.001	Yes	0.83	0.78	0.69
SSN_C_ – Central	32	<0.001	<0.001	Yes	0.82	0.78	0.68
SSN_C_ – South	51	0.08	0.19	No	0.85	0.78	0.70

θ = 1 represents an initial (pre-decline) *N*_e_ of 500 and θ = 10 an *N*_e_ of 5000. *M*-Ratio values that fall below the *M*-Critical value are considered statistically significant at α = 0.05. Results incorporating population structure in the SSN_C_ are shown on the last 3 lines where H denotes historical samples and C denotes contemporary samples.

The mixed results in the SSN_C_ were clarified after accounting for genetic population subdivision. Both the North and Central SSN_C_ samples showed strong evidence of a recent bottleneck with significant heterozygosity excess tests (p<0.001) and shifted modes. The South SSN_C_ sample showed no evidence of a recent bottleneck in either the heterozygosity excess or shifted mode tests. After accounting for populations subdivision there was still no evidence of a bottleneck in the SSN_C_ using the *M*-Ratio method (Table 3).

### Demographic history

We were unable to obtain consistent results for the demographic change analysis in the NW_H_ due to small sample size (n = 5) and therefore, did not include these results in our analyses. However, results from the other three sample groups consistently indicate that there was a large population decline with current *N*_e_ estimates over 90% lower than the estimates of the ancestral *N*_e_. These results were consistent across a variety of prior distributions and both demographic models (exponential and linear). Bayes factor values were >10 for all models indicating strong evidence for a population decline (Table 4).

**Table 4:** The mode and 90% highest posterior density (in parentheses) of the posterior distributions for the Storz and Beaumont [67] models.

Sample	BF	Scale	*N*_0_	*N*_1_	Time (*T*)
Historical-SSN	10.9	**Exp**	**154 (1–2160)**	**1862 (454–7952)**	**442 (96–25249)**
Historical-SSN	13.5	Linear	102 (1–1993)	1922 (457–7838)	1054 (109–61941)
Contemporary-SSN	36.1	**Exp**	**167 (23–838)**	**1613 (383–7102)**	**1693 (60–23307)**
Contemporary-SSN	65.2	Linear	139 (17–692)	1405 (358–8143)	3134 (160–73610)
Contemporary-NW	41.1	**Exp**	**129 (23–513)**	1698 (288–12302)	**2884 (162–37153)**
Contemporary-NW	45.5	Linear	128 (27–547)	1640 (246–19639)	8549 (373–353012)

The Bayes factor (BF) indicates the strength of evidence for a population decline with values greater than 10 representing very strong support. *N*_0_ and *N*_1_ are the current and ancestral *N*_e_ respectively. Time (*T*) represents the date of the change in population size from *N*_0_ to *N*_1_.

**Figure 4:**
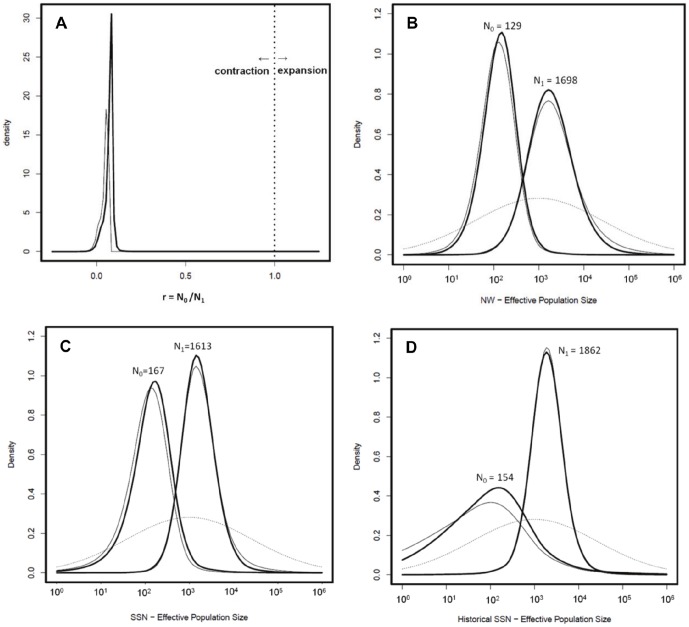
MSVAR results: population size change. A) ratio of current and ancestral population sizes (r = *N*_0_/*N*_1_) where r = 1 signifies population stability, r<1 decline, and r>1 expansion. 4B–D) Posterior distributions of the current (*N*_0_) and ancestral (*N*_1_) effective population size using both the exponential (thick lines) and linear (thin lines) models: B) northwestern-historical, C) southern Sierra Nevada-contemporary, and D) southern Sierra Nevada-historical. The dotted line shows the prior distribution for *N*_0_ and *N*_1_.

The ratio of the posterior distributions of current and ancestral population sizes (r = *N*_0_/*N*_1_) indicates the direction of demographic change where r = 1 signifies population stability, r<1 population decline, and r>1 population expansion. Combining all simulations for all data sets for the exponential model we found the 90% highest posterior density (HPD) of the ratio r to be 0.011–0.095 with a mode of 0.081, and for the linear model an HPD of 0.010–0.066 with a mode of 0.062. These r values indicate that the current *N*_e_ is estimated to be less than 10% of the ancestral *N*_e_ and show an unambiguous signal of population decline for fisher in California (Fig. 4A).

**Figure 5:**
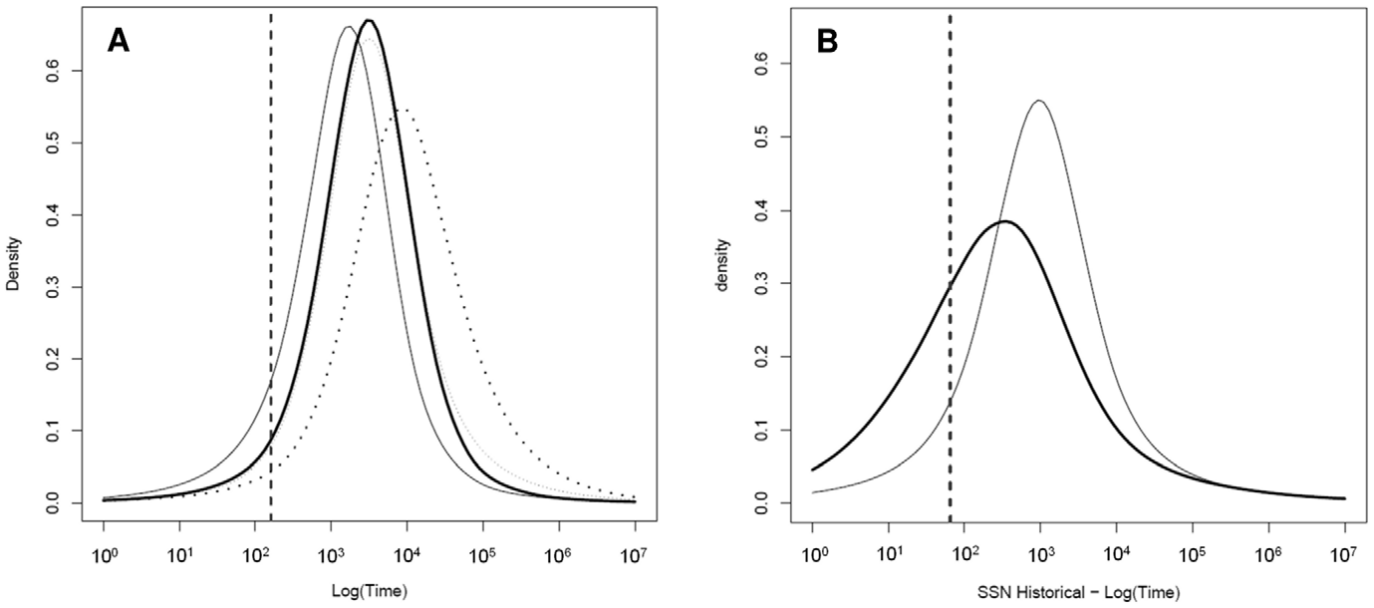
MSVAR results: time of population decline. Posterior distribution of time of decline (*T*) for the linear (thin line) and exponential (thick line) models. A) Time (in years before present) for the contemporary SSN (solid lines) and NW (dashed lines). B) Time for the historical SSN. The vertical dotted line shows the approximate time of the European settlement of California (∼1850) relative to the age of each of the samples.

The modes of the 90% HPD of the posterior distributions for ancestral effective population size (*N_1_*) for the exponential model were SSN_H_  = 1862, SSN_C_ = 1613, and NW_C_ = 1698 compared to modal values for current effective population sizes (*N*_0_) of 154, 167, and 129 respectively (Table 4, Fig. 4 B–D). Estimates for *N*_0_ and *N*_1_ were similar but slightly lower for the linear model. Estimates of the time of population contraction varied between populations, but all showed support for population decline occurring well prior to the European settlement of California (*T*- SSN_C_ = 1693 years before present [YBP], *T*-NW_C_ = 2884 YBP, *T*-SSN_H_ = 442 YBP). We adjusted the time estimates for the SSN_H_ data to reflect the increased age of samples by adding the average age of the sample (95 years) to the estimate. Estimates for the timing of the decline were longer for the linear model than for the exponential model for all sample groups (Table 4, Fig. 5). We put more emphasis on the results of the exponential model because it is likely more realistic when modeling population dynamics [66].

Population subdivision can also bias demographic history models by creating a spurious signal of population decline. The potential bias is greatest for highly subdivided populations (high *F*_ST_), highly variable markers, and species with large *N*_e_ [78]. The recommended ad hoc method to counteract any potential bias created by population subdivision is to sample equally across demes [78]. We followed this ad hoc approach by conducting the MSVAR analysis in the SSN_C_ with numerous samples from all three of the identified demes such that each of the North, Central, and South groups were well represented in our sample. Considering the characteristics of the data used in this analysis (moderate *F*_ST_ values, low variability markers, and small population size) reduce the potential for biased results, combined with our use of the ad hoc method of sampling across demes, we feel our results are robust to the potential bias created by population subdivision.

## Discussion

### Population contraction and isolation

Our analyses supports the hypothesis that the NW and SSN fisher populations became isolated far before the European settlement of California and that the absence of fisher in the northern Sierra Nevada is likely a long standing gap in this species' historical range. We found a genetic signal for a more than 90% reduction in *N*_e_ of fisher and estimated that this decline occurred over a thousand years ago. A decline of this magnitude is consistent with a major range contraction. There is a positive correlation between changes in abundance and distribution, where species' abundance decreases its range also decreases [79]–[82]; species with the strongest declines exhibit the largest range contractions [79]. While the positive correlation between abundance and range size is not universal [79], [83], the extreme decline in *N*_e_ detected in our analyses makes the idea of concurrent stability in range size unlikely. While the 90% highest posterior density of 3 of the 6 models did not definitively exclude a post-settlement decline (Table 4), the vast majority of the mass of the distribution of the time parameter (*T*) support pre-European settlement, with an average of 90% of the contemporary and 81% of historical MCMC chains indicating a time of contraction prior to 1850.

In addition to an ancient population contraction that isolated the SSN from the NW, our analyses indicate the SSN has also undergone a more recent population bottleneck likely associated with the impact of human development in the late 19^th^ and early 20^th^ century. The presence of a bottleneck signal only in the north and central portions of the SSN_C_ and not in the south reflects differences in the extent of anthropogenic influence across the Sierra Nevada. The majority of human settlement, and its associated impacts, occurred in the central and northern Sierra Nevada. Settlement in the southern Sierra was minimal in comparison due to the absence of gold deposits and steeper topography that restricted access to forest lands. Our results indicate that the area at southern tip of the Sierra Nevada may have acted as a refuge for fisher during the era of extensive logging and development that began with the gold rush and continued into the first half of the twentieth century [84]. This area appears to have maintained a stable population size while fisher in the rest of SSN was in decline.

The window of time that the heterozygosity excess and shifted mode tests can detect a bottleneck is shorter than the timeframe for the *M*-Ratio test. The magnitude of the reduction in the *M*-Ratio from equilibrium values is also highly dependent on the pre-bottleneck population size. Accordingly, simulation studies have shown the *M*-Ratio test performs well if the pre-bottleneck population size was large, the bottleneck was of long duration, or the population had time to recover [85]. The length of time that the *M*-Ratio is informative can vary considerably (125–500 generations) depending on the bottleneck characteristics in terms of severity, duration, and post-bottleneck recovery. Assuming a generation interval for fisher of 5 years, significantly reduced *M*-Ratios would be indicative of decline that occurred anywhere from 625–2500 years ago. However, in permanently reduced populations the *M*-Ratio will recover over time, whereas allelic diversity does not [63]. Consequently, a population with low allelic diversity but a high *M*-Ratio, such as was observed in this study, is indicative of a population that has been small for a very long time. This conclusion is further supported by the fact that we found all sample groups to have low genetic diversity, and did not find any significant difference in diversity between contemporary and historical samples (collected between 1880 and 1920). This suggests that a population reduction, and its concurrent reduction in genetic diversity, occurred prior to the dates of the historical samples.

Our data suggests continual isolation of the NW and SSN populations during the last century. The increase in *F*_ST_ from 0.10 in the early 1900s to 0.37 in 2006–2009 shows the genetic isolation of the populations during the intervening years. However, the *F*_ST_ estimates between historical NW and SSN samples are likely biased considering the number of samples available from each population was small and from a relatively limited geographic subset of each area. Genotypic differentiation was strong across all spatial and temporal samples, and the amount of within population genetic differentiation over time period was similar in both areas (*F*_ST_: SSN_H_-SSN_C_ = 0.17, NW_H_-NW_C_ = 0.20) which can be attributed to the effects of genetic drift in small populations over time.

### Considerations for bottleneck tests

Recent studies have found that bottleneck detection methods sometimes perform poorly at detecting very recent or weak population declines [68], [86], [87]. This creates a concern that a post-settlement decline would not be detected even if it had occurred. Girod et al. [68] used simulations to evaluate the ability of MSVAR to detect expansion/declines assessing performance using Bayes factors. Their analyses of populations with recent and/or weak declines resulted in very low Bayes factors (≤3) indicating no support for the detection of a decline. Accordingly, if the decline in the California fisher population was very recent we would expect MSVAR to produce a model with little support (low Bayes factors) reflecting the supposed poor ability of the method to detect recent declines. However, our MSVAR analyses produced high Bayes factors (≥10) for all models showing strongly supported signals of decline. Such high Bayes factors are in agreement with the results of the Girod et al. [68] for more ancient times of contraction (≥50 generations). The poor performance of the heterozygosity excess and *M*-Ratio tests detected in the Girod et al. [68] study is likely due to their simulation being conducted under a strict stepwise mutational model which has been identified as an unrealistically conservative model for microsatellite loci that may not have much power to detect bottlenecks that have actually occurred [88]. Other studies have shown these two methods to have a much higher power to detect bottlenecks [85], [88].

An important consideration in the interpretation of bottleneck tests is the potential influence of isolation by distance (IBD) within populations. While the SSN_C_ has been found to exhibit a significant isolation by distance pattern across the entire population, tests for IBD were non-significant within each of the North, Central, and South subpopulations (J.M. Tucker unpublished data). The clustered distribution of samples in the NW_C_ and SSN_H_ and the small sample size of the NW_H_ prevented us from testing for IBD in these populations. However, IBD has been found to have little effect on the heterozygosity excess method implemented in BOTTLENECK [89]. IBD does influence the *M*-Ratio such that both equilibrium and post bottleneck values of *M* are depressed compared with a non-IBD population. Thus, IBD can result in M values in non-bottlenecked populations that are lower than the Garza and Williamson's [63] recommended *M*-Critical cutoff value of 0.68 providing a false signal of a bottleneck. However, given the consistently high *M* values detected in this study (M = 0.82–0.92, Table 3) we do not feel that IBD biased our *M*-Ratio analyses.

### Effective population size estimates

The similarity between the estimates of *N*_e_ in the NW_C_ and SSN_C_ populations is surprising given that the NW_C_ is thought to have a larger total population size (*N*) than the SSN_C_. There are no published estimates of *N* in the NW_C_, but unofficial estimates place it at between 1000–2000 individuals (C. Carroll personal communication cited in [90]) compared to estimates of 160–360 for the SSN_C_ [91]. The ratio of *N*_e_/*N* is not well understood and can vary considerably between populations or species due to factors such as fluctuating population size, variance in reproductive success, unequal sex ratio, or population density [92]–[95]. Predicted values of the *N*_e_/*N* ratio in the literature vary widely; Nunney [96] estimated that theoretically the *N*_e_/*N* ratio should be 0.5, Nunney and Elam [97] found the average ratio across empirical data from 13 species to be 0.73, and Frankham [92] found the mean ratio across 102 species to be 0.11. Consequently, it is difficult to interpret what the estimated values of *N*_e_ mean in terms of *N* in relation to each population. Extrapolating the modal values of the exponential model for *N*_0_ across a wide range of possible *N*_e_/*N* ratio values of 0.05–0.5, the total population size for the NW could range from 258–2850 and SSN from 334–3380. Both of these population size ranges encompass the current possible estimates of *N* for both areas.

### Biogeographical influences

The population contraction detected in this study and in the ancient mitochondrial divergence date reported by Knaus et al. [24] may reflect a shift in habitat distribution or community composition associated with one of a number of potentially significant climate shifts during the end of the Pleistocene or Holocene epochs. There are many well-known hypotheses about the cause of the mass extinctions and major shifts in species distribution that occurred at the end of the Pleistocene including temperature increases, changes in precipitation, or shifts in the ecological balance due to the arrival of human hunters in North America [98]. In more recent climactic history there are two well documented “mega-droughts” that occurred in California that have not been matched in severity or duration since. These droughts were first described by Stine [99] and were estimated to have lasted over 200 and 140 years each from 832–1074 and 1122–1299 AD respectively [100]. These droughts fall into a period of warmer temperatures referred to as the Medieval Warm Period [101] or Medieval Climate Anomaly [99]. While the divergence dates reported by Knaus et al. [24] would support a late Pleistocene climate shift as a possible cause of the divergence of California fisher populations, the results of this study found dates that support a more recent event, such as the aforementioned mega-droughts as a potential cause of the population contraction. Neither method allows for precise dating of the demographic shift. Nevertheless, both studies show that the contraction of the fisher populations in California pre-dated the gold rush and was not a direct result of the European settlement of California.

The reason fisher would be absent from the central and northern Sierra is perplexing, considering that there is no obvious geographic feature that marks a significant break in the topography or vegetative composition of the Sierra Nevada. However, a number of other species such as the great gray owl (*Strix nebulosa*), wolverine (*Gulo gulo*), and foxtail pine (*Pinus balfouriana*) have also been found to have long term genetic and geographic isolation in the southern Sierra Nevada [11], [102], [103] indicating that there are perhaps unique vegetative, climactic, or topographic elements in this region that are absent from the northern Sierra Nevada. A recent climate assessment has shown the southern Sierra Nevada to be somewhat resistant to climate changes observed elsewhere in California due to the extreme elevation of the mountains in this region [104].

The Sierra Nevada is characterized by a gradual change in its maximum elevation and average slope, such that the elevation of the Sierran crest and average slope is highest in the south. The area of the Sierra Nevada occupied by fisher is at the southernmost extent of its range where the weather is hotter and drier than in other areas. To mitigate the effects of high heat and low humidity, fisher may use cool and damp microhabitats characterized by dense canopies, large diameter trees, steep slopes, and close proximity to water [30]. One possible explanation for fisher presence in the southern Sierra is that the steep topography in this portion of the mountain range facilitates the creation and persistence of these essential microhabitat areas.

Relatively high amounts of subdivision have been reported in other parts of the fisher's range. Kyle et al. [105] found the amount of genetic subdivision observed between fisher populations (global *F*_ST_ = 0.137) was much higher than for other closely related carnivore species of American marten (*F*_ST_ = 0.0198) or wolverine (*F*_ST_ = 0.0427) [106], [107]. A linear regression of genetic versus geographic distance found that fisher have twice the subdivision per unit distance than martens and 5 times more per unit distance than wolverine [108]. The high amounts of subdivision observed in fisher may result from being habitat specialists which makes them especially vulnerable to habitat fragmentation [109], [110]. Strikingly, this study found the structure per unit distance between the SSN_C_ and NW_C_ to be to be ∼10 times greater (0.961/1000 km) and between then SSN_H_ and NW_H_ to be ∼4 times greater (0.348/1000 km) than Kyle et al. [105] found for fisher populations across North America (0.092/1000 km). However, high subdivision is not universal among fisher populations. Populations in southern Ontario, Canada have been found to have weak subdivision and high genetic connectivity attributed to high amounts of gene flow along expansion fronts in a growing population [16], [111].

### Conservation Implications

Our results provide a historical perspective for contemporary conservation and management decisions for fisher in California. There are ongoing debates as to whether efforts should be made to restore connectivity between the NW and SSN and thereby increase genetic diversity in the isolated SSN. The results of this study show that both populations have persisted in isolation far prior to the European settlement of California. Therefore, attempting to restore connectivity between them would be inconsistent with the historical record and run the risk of losing local adaptations that evolved in each population [36]. Given their long term isolation, the NW and SSN fisher populations should be considered independently for management and conservation decisions.

In 2004, the west coast population of fisher (southern Oregon, northwestern California, and southern Sierra Nevada of California) was found warranted but precluded for listing as a single distinct population segment (DPS) under the federal Endangered Species Act [112]. Among the criteria for considering a population as a DPS it must be markedly separated from other populations of the same taxon (discrete) and differ from other populations in its ecological setting or genetic characteristics (significant) [113]. As both of these criteria can be met by quantitative measures of genetic discontinuity or genetic uniqueness [113], the detection of long term genetic isolation of the southern Sierra Nevada fisher population has important implications for its legal status. The observed genetic differentiation coupled with observed differences in diet, home range size, and habitat associations between the SSN and NW [29], [114], [115] speaks to the potential of the SSN population itself as a DPS.

## Supporting Information

Table S1 **Location and collection date of historical fisher genetic samples.** Samples were collected from the Smithsonian National Museum of Natural History (SNM) and the Museum of Vertebrate Zoology at the University of California, Berkeley (MVZ). Samples that successfully genotyped at a minimum of 7 of 10 microsatellite loci are shown in bold.
(DOCX)

Table S2 **Prior and hyperprior parameters for runs of the Storz and Beaumont (2002) analysis implemented in MSVAR.** Columns 3–6 show the starting values for the mean and variance of the prior distributions. Columns 7–10 show the means and variances (and their means and variances) of the hyperprior distributions. Parameters listed are generation interval (g), current *N*_e_ (*N*_0_), ancestral *N*_e_ (*N*_1_), mutation rate scaled in terms of current population size (*θ*), and time (*T*). All values are in a log_10_ scale.
(DOCX)
