# Supplementary material for: Historical and Contemporary DNA Indicate Fisher Decline and Isolation Occurred Prior to the European Settlement of California
Source: PLoS One. 2012 Dec 26;7(12):e52803. doi: 10.1371/journal.pone.0052803 (PMC3530519; doi:10.1371/journal.pone.0052803)
Supplement: Table S1 — Location and collection date of historical fisher genetic samples. Samples were collected from the Smithsonian National Museum of Natural History (SNM) and the Museum of Vertebrate Zoology at the University of California, Berkeley (MVZ). Samples that successfully genotyped at a minimum of 7 of 10 microsatellite loci are shown in bold. (DOCX) [file pone.0052803.s001.docx]

**Table S1: Location and collection date of historical fisher genetic samples.** Samples were collected from the Smithsonian National Museum of Natural History (SNM) and the Museum of Vertebrate Zoology at the University of California, Berkeley (MVZ). Samples that successfully genotyped at a minimum of 7 of 10 microsatellite loci are shown in bold.

| Population | Collection Date | Catalog Number | Location |
| --- | --- | --- | --- |
| **Northwest** | **5-Feb-1884** | **SNM-14395** | **Shasta County** |
|  | **1-Feb-1897** | **SNM-87080** | **Cassel, Rock Creek Mountains** |
|  | 1-Feb 1897 | SNM-87081 | Cassel, Burney Mountain |
|  | 11-May-1889 | SNM-30624 | Cahto California, 3 mi S of Laytonville |
|  | 11-May-1889 | SNM-24025 | Cahto California, 3 mi S of Laytonville |
|  | 28-Mar-1905 | MVZ-20955 | Eden Valley |
|  | 1-Feb-1910 | MVZ-12902 | Helena |
|  | **20-Feb-1911** | **MVZ-12901** | **Helena** |
|  | 13-Dec-1911 | MVZ-16386 | Head Ray's Gulch, 5 miles S of Cecilville |
|  | **19-Feb-1912** | **MVZ-16531** | **Cecil Lake, 8 miles S of Cecilville** |
|  | **25-Mar-1912** | **MVZ-16596** | **head Black Gulch, 10 miles S of Cecilville** |
|  | 27-Jan-1913 | MVZ-19095 | 8 mi SE of Cecilville |
|  | 23-May-1917 | SNM-227117 | Covelo, California |
|  | 16-Sep-1917 | SNM-227118 | E. Fork Wells Creek, 15 miles E of Hayfork |
|  | not recorded | SNM-21233 | Shasta County |
|  | not recorded | SNM-3415A | Fort Crooks , near Fall River Mills, California |
| **Southern Sierra** | **30-Jan- 1892** | **SNM-32315** | **Big Creek, Mariposa County** |
| **Nevada** | 14-Jan-1893 | SNM-51270 | Big Creek, Mariposa County |
|  | April-1893 | SNM-52821 | Wawona, Yosemite NP |
|  | **23-Dec-1895** | **SNM-81094** | **Atwell's Mill, Sequoia NP** |
|  | 29-Mar-1905 | MVZ-21396 | Grouse Creek, Yosemite NP |
|  | 26-Jan-1911 | SNM-171002 | Yosemite Valley |
|  | 17-Dec-1915 | MVZ-23668 | Chinquapin, Yosemite NP |
|  | **28-Dec-1915** | **MVZ-23883** | **6 miles S of Hetch Hetchy Valley** |
|  | **14-Jan-1915** | **MVZ-23884** | **6 miles S of Hetch Hetchy Valley** |
|  | **20-Jan-1916** | **MVZ-23885** | **6 miles S of Hetch Hetchy Valley** |
|  | 22-Feb-1916 | MVZ-23686 | Near Fort Monroe, Yosemite NP |
|  | **3-Feb-1917** | **MVZ-24740** | **Fort Monroe, Yosemite NP** |
|  | **1-Dec-1918** | **MVZ-29809** | **Grouse Creek, near Wawona Road, Yosemite NP** |
|  | 27-Jan-1919 | MVZ-29810 | Yosemite Valley |
|  | **28-Jan-1919** | **MVZ-29811** | **Grouse Creek, near Wawona Road, Yosemite NP** |
|  | **1-Feb-1919** | **MVZ-29812** | **Tuolumne Big Trees, Yosemite NP** |
|  | **1-Feb-1919** | **MVZ-29813** | **Tuolumne Big Trees, Yosemite NP** |
|  | 1-Jan-1920 | MVZ-31132 | Hog Ranch Ranger Station (Mather), Yosemite NP |
|  | **1-Feb-1920** | **MVZ-31133** | **Near Crane Flat, Yosemite NP** |
|  | **8-Feb-1920** | **MVZ-31129** | **Near Big Meadows, Coulterville Rd, Yosemite NP** |
|  | **14-Feb-1920** | **MVZ-31093** | **Yosemite Valley** |
|  | **14-Feb-1920** | **MVZ-31094** | **Yosemite Valley** |
|  | **1-Apr-1920** | **MVZ-31326** | **Yosemite NP** |
|  | **18-Jan-1919** | **MVZ-29791** | **Yosemite NP** |
