# Supplementary material for: Historical and Contemporary DNA Indicate Fisher Decline and Isolation Occurred Prior to the European Settlement of California
Source: PLoS One. 2012 Dec 26;7(12):e52803. doi: 10.1371/journal.pone.0052803 (PMC3530519; doi:10.1371/journal.pone.0052803)
Supplement: Table S2 — Prior and hyperprior parameters for runs of the Storz and Beaumont (2002) analysis implemented in MSVAR. Columns 3–6 show the starting values for the mean and variance of the prior distributions. Columns 7–10 show the means and variances (and their means and variances) of the hyperprior distributions. Parameters listed are generation interval (g), current N e (N 0), ancestral N e (N 1), mutation rate scaled in terms of current population size (θ), and time (T). All values are in a log10 scale. (DOCX) [file pone.0052803.s002.docx]

**Table S2: Prior and hyperprior parameters for runs of the Storz and Beaumont (2002) analysis implemented in MSVAR.** Columns 3-6 show the starting values for the mean and variance of the prior distributions. Columns 7-10 show the means and variances (and their means and variances) of the hyperprior distributions. Parameters listed are generation interval (g), current *N*_e_ (*N*_0_), ancestral *N*_e_ (*N*_1_), mutation rate scaled in terms of current population size (*Ѳ)*, and time (*T)*. All values are in a log_10_ scale.

| Run# | g | log(*N_0_*) | log(*N_1_*) | log(*θ*) | log(*T*) | log(*N_0_*) | log(*N_1_*) | log(*θ*) | log(*T*) |
| --- | --- | --- | --- | --- | --- | --- | --- | --- | --- |
| 01 | 5 | 4 1 | 4 1 | -3.3 1 | 3 1 | 3 2 0 0.5 | 3 2 0 0.5 | -3.3 0.25 0 0.5 | 3 2 0 0.5 |
| 02 | 5 | 4 1 | 4 1 | -3.3 1 | 4 1 | 3 2 0 0.5 | 3 2 0 0.5 | -3.3 0.25 0 0.5 | 3 2 0 0.5 |
| 03 | 4 | 4 1 | 3 1 | -3.3 1 | 4 1 | 3 2 0 0.5 | 3 2 0 0.5 | -3.3 0.25 0 0.5 | 3 2 0 0.5 |
| 04 | 5 | 3 1 | 3 1 | -3.3 1 | 4 1 | 2 2 0 0.5 | 2 2 0 0.5 | -3.3 0.25 0 0.5 | 3 2 0 0.5 |
| 05 | 5 | 4 1 | 4 1 | -3.3 1 | 4 1 | 4 3 0 0.5 | 4 3 0 0.5 | -3.3 0.25 0 0.5 | 3 2 0 0.5 |
| 06 | 5 | 4 1 | 4 1 | -3.3 1 | 2 1 | 3 2 0 0.5 | 3 2 0 0.5 | -3.3 0.25 0 0.5 | 2 2 0 0.5 |
